# Linking Personality Traits to Individual Differences in Affective Spaces

**DOI:** 10.3389/fpsyg.2020.00448

**Published:** 2020-03-12

**Authors:** Seth M. Levine, Aino L. I. Alahäivälä, Theresa F. Wechsler, Anja Wackerle, Rainer Rupprecht, Jens V. Schwarzbach

**Affiliations:** ^1^Department of Psychiatry and Psychotherapy, University of Regensburg, Regensburg, Germany; ^2^Department of Clinical Psychology and Psychotherapy, University of Regensburg, Regensburg, Germany

**Keywords:** affective science, Big Five, clustering, emotions, individual differences, personality

## Abstract

Different individuals respond differently to emotional stimuli in their environment. Therefore, to understand how emotions are represented mentally will ultimately require investigations into individual-level information. Here we tasked participants with freely arranging emotionally charged images on a computer screen according to their subjective emotional similarity (yielding a unique affective space for each participant) and subsequently sought external validity of the layout of the individuals’ affective spaces through the five-factor personality model (Neuroticism, Extraversion, Openness to Experience, Agreeableness, Conscientiousness) assessed via the NEO Five-Factor Inventory. Applying agglomerative hierarchical clustering to the group-level affective space revealed a set of underlying affective clusters whose within-cluster dissimilarity, per individual, was then correlated with individuals’ personality scores. These cluster-based analyses predominantly revealed that the dispersion of the negative cluster showed a positive relationship with Neuroticism and a negative relationship with Conscientiousness, a finding that would be predicted by prior work. Such results demonstrate the non-spurious structure of individualized emotion information revealed by data-driven analyses of a behavioral task (and validated by incorporating psychological measures of personality) and corroborate prior knowledge of the interaction between affect and personality. Future investigations can similarly combine hypothesis- and data-driven methods to extend such findings, potentially yielding new perspectives on underlying cognitive processes, disease susceptibility, or even diagnostic/prognostic markers for mental disorders involving emotion dysregulation.

## Introduction

Emotions are an aspect of everyday life, and questions regarding their elusive nature have been of interest for millennia. While recent discussions (Hamann, 2012; Lindquist et al., 2012; Adolphs, 2017; Barrett, 2017) and neuroscientific investigations (Grimm et al., 2006; Nielen et al., 2009; Baucom et al., 2012; Goodkind et al., 2012; Chikazoe et al., 2014; Shinkareva et al., 2014; Kragel et al., 2016; Saarimäki et al., 2016) have advanced the field of affective (neuro)science, there still remains no consensus concerning the inherent features of emotions (Ekman, 2016). Alongside this general interest in understanding affective functionality is the clinical interest in understanding affective dysfunctionality (Gross and Jazaieri, 2014), as emotion dysregulation has been associated with various mental disorders (Mennin et al., 2005; Reimherr et al., 2005; Etkin and Wager, 2007; Amstadter, 2008; Taylor et al., 2012; Carpenter and Trull, 2013). Following the drive for improved translational research in psychiatry (Machado-Vieira, 2012; Knüppel et al., 2013) and personalized medicine (Hamburg and Collins, 2010), we sought to study individual differences in affective information representations by combining data-driven analyses of a behavioral experiment with psychological measures of personality traits, given the well-established link between emotions and personality (Costa and McCrae, 1980; Gross et al., 1998; Kokkonen and Pulkkinen, 2001; Ng and Diener, 2009).

To this end, we employed the multi-arrangement method (Goldstone, 1994) and inverse multidimensional scaling (Kriegeskorte and Mur, 2012), which have recently been utilized in the domain of cognitive neuroscience (Mur et al., 2013; Charest et al., 2014; Bracci et al., 2016; Levine et al., 2018c), to emotionally charged stimuli in order to discern individualized structures (that reflect mental representations) of affective information. Participants freely arranged the stimuli according to their subjective emotional similarity in a continuous space, which resulted in a label-free, unique representation of an “affective space” for each participant. Other recent work has investigated the organizing principles of affect/emotions using psychological (Nummenmaa et al., 2014, 2018; Koch et al., 2016; Cowen and Keltner, 2017; van Tilburg and Igou, 2017) and neuroscientific (Kragel and LaBar, 2015; Skerry and Saxe, 2015; Saarimäki et al., 2018) methods; here we took an approach that focused on how individuals differ in terms of underlying properties of their affective spaces. Being able to distinguish individuals based on affective information (Hamann and Canli, 2004) – and subsequently determine normal variability using a combination of hypothesis-driven and data-driven methods – may offer new routes for social psychology/neuroscience to inform the clinical realm (Cacioppo et al., 2014).

The current study employed data-driven hierarchical clustering to identify clusters of stimuli underlying the affective space. However, to avoid blindly applying unsupervised machine learning methods to the high-dimensional affective spaces, we externally validated the individual differences in affective clustering with differences in personality traits from the five-factor model [“Big Five”: Neuroticism, Extraversion, Openness to Experience, Agreeableness, and Conscientiousness (McCrae and Costa, 1985)], assessed by the NEO-Five Factor Inventory [NEO-FFI (Borkenau and Ostendorf, 2008)]. As prior neuroimaging work has shown that the layout of a representational space can be meaningfully altered by attentional or task-related processes (Brouwer and Heeger, 2013; Nastase et al., 2017) and recent psychological work has shown that clusters within an affective space map onto certain aspects of emotion information (Nummenmaa et al., 2018), we specifically sought to determine whether the *within-cluster distance* of individuals’ affective clusters [which can be seen as the cohesiveness of the concept(s) underlying the pertinent cluster (Iordan et al., 2015)] was linked to participants’ personality traits. The idea of investigating emotions as they relate to personality follows from decades of evidence that personality is tied to internal or attentional biases of emotion-related processing (Richards et al., 1992; Derryberry and Reed, 1994; Most et al., 2006; Thomsen et al., 2014). Moreover, classic theories of personality would predict that Neuroticism associates with negative information and Extraversion associates with positive information (Eysenck, 1967; Costa and McCrae, 1980; Larsen and Ketelaar, 1989; Rusting and Larsen, 1997). Exploring the relationship between personality and emotional similarity (here, operationalized as cluster dispersion within affective spaces) will allow researchers to investigate how underlying cognitive processes, such as attention or executive control, may interact with personality traits to drive healthy and unhealthy behavior, and whether such interactions are tied to specific brain regions/networks. The findings we present corroborate and extend current knowledge regarding the relationship between affect and personality, validate the non-spurious structure of individualized affective spaces revealed by data-driven methods, and open the door for future research to translate such data-driven paradigms to the clinical domain.

## Materials and Methods

### Participants

One hundred one participants (36 males, 65 females; mean (± σ) age = 24.2 (± 2.59) years) were recruited from the local community via information posters. Participants reported neither a current diagnosis of neurological or mental disorders nor the intake of any psychotropic medication, provided written informed consent before taking part in the study, and were monetarily compensated for their time after completing the experiment. As filling out the NEO-FFI questionnaire was a follow-up procedure to the behavioral experiment, only participants who had initially agreed to be contacted for future studies were asked for their participation in completing the NEO-FFI questionnaire. Of 77 participants contacted, 58 participants (15 males, 43 females; mean (± σ) age = 25.4 (± 2.75) years) ultimately completed the questionnaire, five of whom were compensated with bookstore gift cards following a raffle. All experimental procedures complied with the Declaration of Helsinki and were approved by the local ethics committee at the University of Regensburg.

### Apparatus

The experiment was conducted on a 27” Apple iMac using MATLAB R2015b (The MathWorks, Natick, MA, United States). The MATLAB code used to run the experiment was adapted from that described by Kriegeskorte and Mur (2012). All analyses were carried out in MATLAB and SPSS ver. 25 (IBM Corp., Armonk, NY, United States).

### Stimuli

Images used in the experiment were obtained from the International Affective Picture System (IAPS) database (Lang et al., 2008), which contains images with standardized valence and arousal ratings that can be roughly divided into nine categories: animals, people, nature, food, household items, erotic images, accidents, violence, and war. We pseudo-randomly chose six images per category for the experiment, yielding a stimulus set comprising 54 images. If the computer algorithm chose two images that were very visually similar (e.g., two pictures of a dog in a similar position, or two pictures of knives on a table), we manually selected a replacement image with the same (or very similar) valence and arousal values from the corresponding category (see Supplementary Table 1 for the corresponding image ID’s and their respective valence and arousal ratings). Creating the stimulus set was ultimately a trade-off between improved sampling of the emotion space and total experiment duration, as the distance between each pair of images must be computed; the total number of pairwise distances in our case is given by the binomial coefficient 54 choose 2 (i.e., 1,431), which yielded a total experiment duration of, on average, slightly more than one hour per participant.

### Trial Protocol

The experiment followed the protocol laid out by Kriegeskorte and Mur (2012), in which participants arrange images on a two-dimensional circular “arena” on a computer screen according to a particular organizing principle. We asked participants to arrange pictures according to how they felt when they viewed each image. That is, images evoking similar emotions should be placed closer together while images evoking dissimilar emotions should be placed further apart; thus the distance between images reflects their relative emotional dissimilarity for the participant.

**FIGURE 1:**
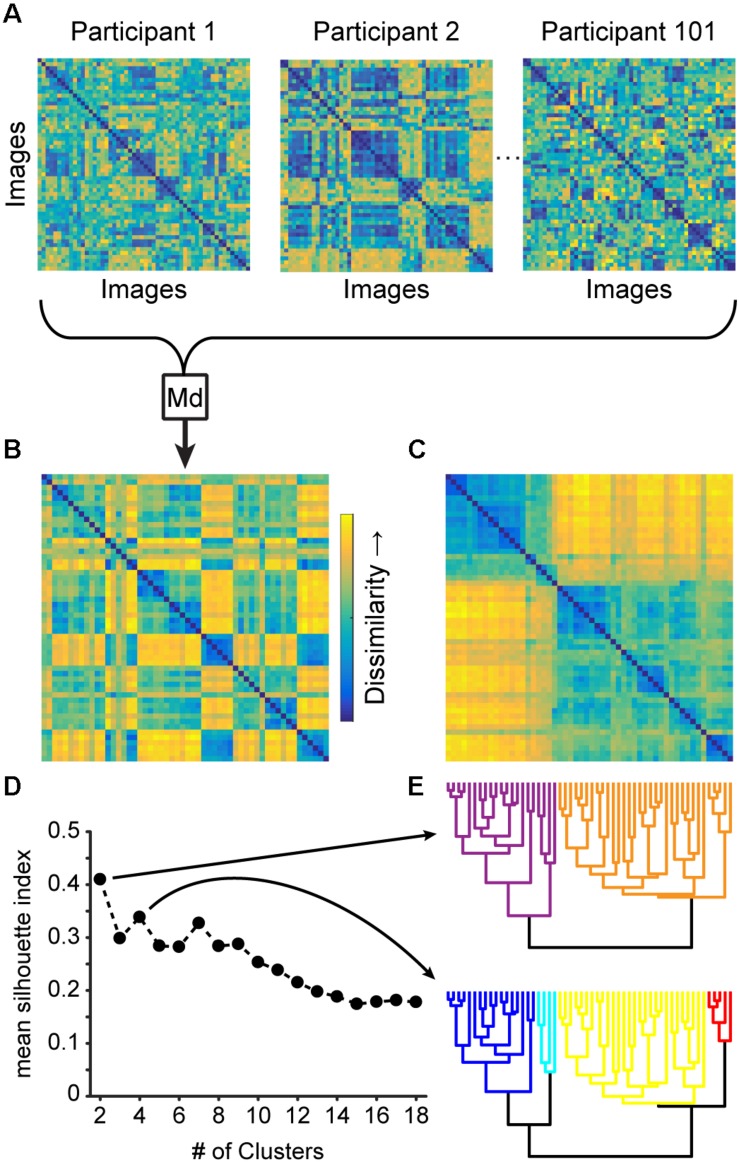
**(A)** Having organized the 54 images on a computer screen based on the emotional similarity of the stimuli, a representation of each participant’s affective space was obtained via inverse multidimensional scaling, which can be visualized as a dissimilarity matrix depicting the distance between each pair of stimuli. **(B)** Computing the median (Md) of all 101 participant’s normalized affective spaces yielded a single group-median affective space, which **(C)** we reorganized based on **(E)** agglomerative hierarchical clustering. The clustering was validated with **(D)** the Silhouette index, which revealed 2- and 4-cluster solutions (as noted by the upticks, highlighted by the arrows). **(E)** The dendrograms underlying both clustering solutions are color-coded with warm colors reflecting the more positively valenced clusters and cool colors reflecting the more negatively valenced clusters (see section “Hierarchical Clustering”).

No more than ten images were shown during a given trial to improve visibility of the pictures (i.e., fitting 54 pictures on the screen at the same time would render them all so small that their content would become unrecognizable). Additionally, we implemented a zoom function, which allowed participants to see the fine-grained detail of each picture. When the participant finished organizing the images, the next trial began with another set of images, for which there remained the least amount of evidence regarding their relative distances to the other images [the lift-the-weakest algorithm (Kriegeskorte and Mur, 2012)]. Thus, some of the images may have been present in two or more successive trials, and these particular images shown on a given trial differed between individuals, depending on how they organized the images throughout the course of the experiment. After distances for all 1,431 pairwise dissimilarities were obtained, inverse multidimensional scaling transformed the two-dimensional distances on the computer screen into a 54 × 54 dissimilarity matrix (DSM; Figure 1A), which represents the high-dimensional dissimilarity structure of the item arrangements (Kriegeskorte and Mur, 2012). However, because the DSM is symmetric across the main diagonal, each participant can be represented by a 1,431-element vector, obtained by vectorizing either the upper or lower triangle of the DSM. We will hereinafter refer to the vector representation of a DSM as a dissimilarity vector (DSV).

### NEO-FFI

We used the German version of the NEO-FFI (Borkenau and Ostendorf, 2008) to measure the Big Five personality factors Neuroticism, Extraversion, Openness to Experience, Agreeableness, and Conscientiousness. For each participant, the sum scores for all five factors were assessed and then transferred into a gender- and age-related T-score based on the reference samples reported by Borkenau and Ostendorf (2008), which expresses the manifestation of a Big Five personality factor in an individual with respect to the manifestation in a gender- and age-corresponding population. For a list of the resulting NEO-FFI scores and their descriptive statistics, see Supplementary Table 2.

### Data Analysis

#### Hierarchical Clustering

Because all participants’ affective spaces clustered slightly differently, we wanted to ensure that we were comparing clusters across participants based on the same stimuli. To this end, we first scaled each participant’s DSV to a range of [0–1] by dividing each DSV by its maximum dissimilarity value. We then computed the median of all 101 scaled DSVs and carried out agglomerative hierarchical clustering using complete linkage (Lance and Williams, 1967) on the group-median DSV (Figure 1B and Supplementary Figure 1).

We validated the stability of different numbers of clusters underlying the group-median affective space by iteratively cutting the resulting dendrogram at different heights and calculating the silhouette index (Rousseeuw, 1987). This index measures how well a given data point is assigned to a particular cluster (i.e., within-cluster distance) compared to the nearest-neighbor cluster (i.e., between-cluster distance) on a scale from −1 to 1), with higher values indicating a more appropriate clustering solution (n.b., group-median clusters produced higher silhouette values than group-mean clusters). This procedure yielded a 2-cluster and 4-cluster solution (Figures 1C–E), which we then mapped back onto each image pair from the participants’ individual DSVs, allowing us to calculate, for each participant, the median (rather than mean, as the distance data tended to be skewed) within-cluster distances (which we normalized by the number of elements in the respective cluster). Formally, the median within-cluster distance *D* for cluster *c* was calculated as $\frac{m\,e\,d\,i\,a\,n\,\left(D_{c}\right)}{\left|c\right|}$, where |*c*| denotes the cardinality of cluster *c*.

**FIGURE 2:**
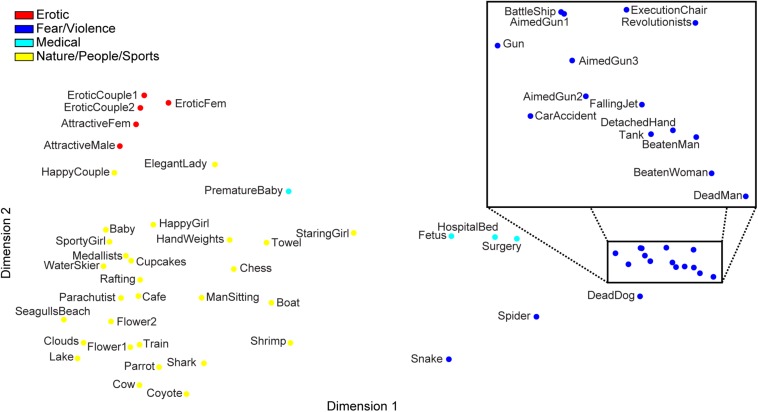
The 54 IAPS stimuli used in the experiment plotted in a two-dimensional space obtained from classical multidimensional scaling of the group-median affective space. The clusters are color-coded according to the 4-cluster solution from the hierarchical clustering (see Figure 1). The 2-cluster solution simply incorporates the cool colors into one cluster and the warm colors into the other.

Following classical multidimensional scaling of the group-median affective space (see Figure 2 for ease of data visualization only, as the actual cluster distances were computed directly from participants’ DSVs), the first dimension (variance explained (VE) = ∼48%) corresponded to the IAPS valence values (Pearson’s *r* = 0.94, *p* = 8.5 × 10^–25^), when partialing out the arousal values; as such, we assigned the labels of “positive” and “negative” to the resulting clusters from the 2-cluster solution. To a far lesser degree, the second and third dimensions corresponded to the IAPS arousal values (*r* = 0.38, *p* = 0.005, VE = ∼8%; *r* = 0.50, *p* = 1.5 × 10^–4^, VE = ∼5%, respectively), while partialing out the valence values. To aid in interpreting the results of the subsequent multiple regression (see next section), we ascribed the following descriptive labels to the four clusters based on the general contents of the clusters: “erotic” (positive sub-cluster), “fear/violence” (negative sub-cluster), “medical” (negative sub-cluster), “nature/people/sports” (positive sub-cluster).

#### Multiple Linear Regression

For each affective cluster (in each clustering solution), we carried out multiple regression in order to assess the degree to which the Big Five personality factors predicted the median dispersion of the affective clusters. To determine, in an unbiased manner, which regressors were in the optimal model, we took a combinatorial approach to multiple regression. Specifically, given the five factors (Neuroticism, Extraversion, Openness, Agreeableness, Conscientiousness), there were 2^5^ – 1 possible models (i.e., $\sum_{k=1}^{5}\left(\begin{array}{c} 5\\ k \end{array}\right)$, including only the linear terms), each containing a different combination of the five factors as predictors. To find the optimal model, we performed the regression analysis with each of the 31 models and selected the model with the lowest Bayesian Information Criterion (BIC) (Schwarz, 1978). For each model, the cluster dispersion values and the personality scores were z-scored (using their respective sample means and sample standard deviations) to center the data, and a constant predictor was included. To account for having tested 31 models, we further carried out two Monte Carlo procedures to non-parametrically determine whether the optimal model outperformed models applied to randomized median cluster distances. First, we tested all 31 models on the randomized data and stored the minimum BIC (regardless of the model that yielded this BIC) to control for the inflated family-wise error rate (FWER) from having tested multiple models. Repeating this procedure 1,000 times (per affective cluster) provided us with a null distribution of 1,000 minimum BIC values, from which we calculated empirical *p*-values for the observed optimal BIC (denoted in the results as p_*FWER*_(all): for a comparison against all models when all models were fed random data). Second, we also stored the BIC resulting from the *observed optimal model* when this same model was applied to randomized data 1,000 times. This way, we obtained two empirically derived *p*-values for our optimal model (one from a minimum-BIC null distribution and one from a same-model-BIC null distribution [denoted in the results as p(same): for a comparison against the same model when fed random data]), which indicate how likely it is to find that the optimal model produces a particular BIC under the null hypotheses of no relationship between the Big Five personality factors and the median cluster distances.

## Results

To determine whether the structure of the affective spaces was related to personality dimensions, we sought to predict the individualized median intra-cluster distance (for different affective clusters) from the Big Five personality factors using multiple linear regression. Below are the results from the best-performing regression models for the affective clusters underlying both the 2- and the 4-cluster solutions (see also Supplementary Table 4).

### Two-Cluster Solution: Larger Negative Cluster Dispersion Corresponds to Higher Neuroticism

Starting with the 2-cluster solution from the hierarchical clustering analysis, the optimal model to predict the negative cluster’s dispersion contained the Neuroticism scores as its sole regressor (β = 0.34, SE = 0.126, *t*_56_ = 2.73, *p* = 0.0084), indicating that a higher Neuroticism score tended to predict a larger cluster dispersion. Although this model marginally failed to surpass the statistical threshold when compared to all other models in the Monte Carlo procedure [*p*_*FWER*_(all) = 0.056], it did perform better than chance when fed real data compared to randomized data [*p*(same) = 0.01]. With respect to the positive cluster’s dispersion, while the optimal model contained the Agreeableness scores as its sole regressor (β = −0.22, SE = 0.130, *t*_56_ = −1.65, *p* = 0.1036), this model did not perform better than chance would predict [*p*_*FWER*_(all) = 0.383, *p*(same) = 0.11).

### Four-Cluster Solution: Opposite Dispersion Patterns of the Fear/Violence Cluster for Neuroticism and Conscientiousness

**FIGURE 3:**
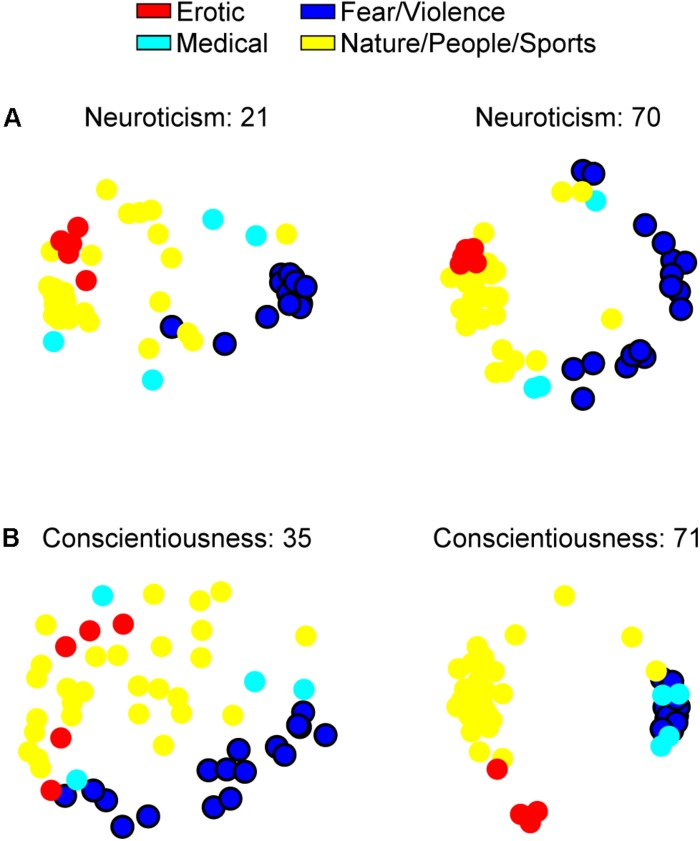
Individualized affective spaces from the participants with the lowest and highest **(A)** Neuroticism and **(B)** Conscientiousness T-scores in our sample mapped onto two dimensions using multidimensional scaling solely to help visualize the effect underlying the multiple regression results, in this case those reported in section “Four-Cluster Solution: Opposite Dispersion Patterns of the Fear/Violence Cluster for Neuroticism and Conscientiousness.” Following the same color-scheme as in the previous figures, circles represent the 54 stimuli, and black contours are drawn around the stimuli that are members of the cluster whose dispersion was predicted by the multiple regression.

With the group-level affective space split into four clusters, the previously negative cluster further subdivided into a cluster whose images encompassed concepts of “fear/violence” and a cluster whose images encompassed “medical” concepts (Figure 2). Our combinatorial multiple regression procedure revealed that the optimal model contained Neuroticism (β = 0.280, SE = 0.127, t_55_ = 2.20, *p* = 0.032) and Conscientiousness (β = −0.289, SE = 0.127, *t*_55_ = −2.28, *p* = 0.0266) as the two regressors to predict the fear/violence cluster dispersion [*p*_*FWER*_(all) = 0.014, *p*(same) = 0.0009; see Supplementary Table 3 for a list of all models that survived the statistical threshold]. Similarly to the results from the 2-cluster analysis, Neuroticism scores tended to increase with increasing cluster dispersion (Pearson’s *r* = 0.381). Conscientiousness scores, however, showed the opposite pattern in that they tended to decrease as cluster dispersion increased (Pearson’s *r* = −0.388; see Figure 3 for a visual depiction of this effect). Additionally, the optimal model to predict the medical cluster’s dispersion contained only Neuroticism as the regressor (β = 0.207, SE = 0.131, *t*_56_ = 1.58, *p* = 0.12), but this model did not perform better than chance would predict [*p*_*FWER*_(all) = 0.424, *p*(same) = 0.11].

### Four-Cluster Solution: Increasing Erotic Cluster Dispersion Predicted by Decreasing Openness, Agreeableness, and Conscientiousness

Just as the negative cluster from the 2-cluster solution was further subdivided in the 4-cluster solution, so was the positive cluster, which yielded an “erotic” cluster and a generic positive cluster (labeled as “nature/people/sports”) containing the remaining images that included mainly images related to sports, people, nature, etc. Interestingly, the optimal model to predict dispersion of the erotic cluster was a three-regressor model comprising Openness (β = −0.280, SE = 0.120, *t*_54_ = −2.34, *p* = 0.023; Pearson’s *r* = −0.253), Agreeableness (β = −0.291, SE = 0.12, *t*_54_ = −2.43, *p* = 0.019; Pearson’s *r* = −0.258), and Conscientiousness (β = −0.314, SE = 0.120, *t*_54_ = −2.61, *p* = 0.012; Pearson’s *r* = −0.266). Although this model also marginally failed to surpass the statistical threshold when compared to all other models in the Monte Carlo procedure [*p*_*FWER*_(all) = 0.06], it did perform better than chance would predict when fed real data compared to randomized data [*p*(same) = 0.006]. Regarding the generic positive cluster of nature/people/sports images, similar to the 2-cluster solution, the optimal model contained the sole regressor of Agreeableness (β = −0.161, SE = 0.132, *t*_56_ = −1.22, *p* = 0.228), but, once again, this model did not outperform what one would expect from randomness alone [*p*_*FWER*_(all) = 0.72, *p*(same) = 0.24].

## Discussion

In order to explore the underlying structure of individuals’ affective spaces, we conducted an experiment in which participants organized emotionally charged images according to their individualized emotional similarity and combined data-driven analyses with psychological measures of personality traits. With this combination of methods, we specifically sought associations between personality traits of the five-factor model (“Big Five”) and the dispersion of affective clusters [which reflect the cohesiveness of the concept(s) underlying the pertinent cluster (Iordan et al., 2015)] that populated individuals’ affective spaces. Importantly, this approach demonstrated that the clustering of individuals’ affective spaces, obtained through unsupervised machine learning methods, is not spurious, as portions of it can be externally validated with information from individuals’ personality traits.

The primary result that survived our statistical controls came from the 4-cluster solution. Multiple regression revealed that increasing dispersion of the fear/violence cluster was linked to increasing Neuroticism and decreasing Conscientiousness (see also Supplementary Table 5). Such an opposite relationship between these two personality dimensions is not entirely unexpected, as it has been reported as a general finding when assessing the Big Five personality factors (Costa et al., 1991), linked to mental disorders (Trull and Sher, 1994; Kotov et al., 2010), and tied to such domains as social media activity (Liu et al., 2016), emotional problems in adolescents (Smith et al., 2017), and even physiological mechanisms of inflammation (Sutin et al., 2010). Regarding Neuroticism, it is possible that higher Neuroticism coincides with a greater differentiation in the processing of negative stimuli, thereby leading to a finer-grained categorization (i.e., a more dispersed clustering) of negative information. This notion is backed by higher-Neuroticism individuals exhibiting increased processing of unpleasant information (Gomez et al., 2002; Chan et al., 2007) and tending to describe themselves as anxious (McCrae et al., 1986), as anxious individuals selectively attend to negative stimuli (Broadbent and Broadbent, 1988; MacLeod and Mathews, 1988). Regarding Conscientiousness, higher-Conscientiousness individuals have been described as rigorous and orderly (Costa and McCrae, 1992) and have demonstrated greater emotional control when recovering from negative stimuli (Javaras et al., 2012). Relatedly, increased clustering and efficiency of the frontoparietal network (Toschi et al., 2018), which is considered a critical brain network for cognitive control (Miller and Cohen, 2001), has been associated with higher Conscientiousness. As such, higher-Conscientiousness individuals may have more control over (or a greater need for) compartmentalizing negative information and appropriately managing their resulting behavior. Given that the layout of information in individuals’ representational spaces can be altered by processes such as attention (Nastase et al., 2017), typicality judgments (Iordan et al., 2016), and aversive-learning (Dunsmoor et al., 2014; Levine et al., 2018b), it is possible that these personality traits interact with (or even regulate the effectiveness of) such processes to determine how negative information populates individuals’ representational spaces. Thus, the correspondence between personality traits and the degree of differentiation of negative information can be explored as, for example, a biomarker for susceptibility to particular disorders or a further method of classifying disorders involving negative affect.

Our findings also have implications for social and affective neuroscience studies. Recent functional magnetic resonance imaging investigations have employed multivariate pattern analysis (Haxby et al., 2001) in an effort to understand how brain regions or networks represent information related to emotion processing (Bush et al., 2018; Kryklywy et al., 2018; Levine et al., 2018c; Saarimäki et al., 2018). Findings like those we present here can inform such studies by underscoring how the heterogeneity of personality information can influence the distribution of information in affective spaces. As a result, this non-homogeneous affective information across individuals may impact sensitivity in neuroimaging studies of affect, especially those based on longitudinal or pre-post designs, which need to take affective variability into account (Dauvier et al., 2019). This notion is only emphasized by the recent studies that have utilized data from the Human Connectome Project (Van Essen et al., 2013) to link individuals’ personality data to resting-state functional networks (Dubois et al., 2018; Mulders et al., 2018; Nostro et al., 2018; Toschi et al., 2018; Passamonti et al., 2019), as certain aspects of the functions underlying these networks may also vary with differences in personality. In some cases, such heterogeneity (in both healthy and clinical populations) may be exactly the variable of interest when translating findings and methods from cognitive neuroscience to (precision) psychiatry (Feczko et al., 2019).

In terms of limitations, it is possible that specific facets of the personality dimensions underlie our results. However, we cannot accurately relate facet-level information to particular aspects of individuals’ affective spaces because we administered the NEO-FFI rather than the NEO-PI-R (Costa and McCrae, 1992), which is a longer five-factor model personality questionnaire allowing researchers to investigate the six distinct facets of each of the personality factors. Our approach is therefore less sensitive to exploring facet-level information, which could be a critical component in further specifying the value of such findings. Thus, future investigations following this direction could make use of more detailed questionnaires. An additional, generic drawback is that the regression analyses reported here come from only 58 participants (∼57% of the total sample), as we did not have questionnaire data from all participants. This reduction in the sample size consequently reduced the statistical power of our analyses (*cf.* Supplementary Figure 2).

## Conclusion

In conclusion, we carried out a behavioral experiment based on label-free, subjective, emotional similarity judgments and combined data-driven analyses with the classical Big Five personality traits to investigate cluster dispersion in individualized affective spaces. Our combination of methods revealed, primarily, a relationship between how individuals tended to judge the emotional distance of negatively charged stimuli and the personality dimensions of Neuroticism and Conscientiousness. These findings demonstrate that there is non-spurious structure underlying affective spaces revealed with unsupervised machine learning methods. Such assumption-free techniques may help bridge affective sciences with the clinical domain by providing objective measures of normal affective variability across individuals and characterizing how such variability may interact with personality traits and relate to mental conditions. Future investigations can scrutinize such findings, for example, for their longitudinal value in identifying subtypes of particular patient populations. Such directions offer clinical psychology and psychiatry the opportunity to adapt psychological and neuroscientific methods to develop new markers for critical issues such as disease susceptibility and treatment response.

## Data Availability Statement

The raw data supporting the conclusions of this article will be made available by the authors, without undue reservation, to any qualified researcher upon reasonable request. A subset of the data presented in this manuscript were previously made public in a preprint at PsyArXiv (Levine et al., 2018a).

## Ethics Statement

The studies involving human participants were reviewed and approved by the Ethics Committee at the University of Regensburg. The patients/participants provided their written informed consent to participate in this study.

## Author Contributions

SL and JS designed the study. RR contributed to the experiment resources. SL, AA, and AW acquired the data. SL and TW analyzed the data. SL, AA, and TW drafted the manuscript. All authors revised and approved the final version of the manuscript.

## Conflict of Interest

The authors declare that the research was conducted in the absence of any commercial or financial relationships that could be construed as a potential conflict of interest.
