# Supplementary material for: Linking Personality Traits to Individual Differences in Affective Spaces
Source: Front Psychol. 2020 Mar 12;11:448. doi: 10.3389/fpsyg.2020.00448 (PMC7082752; doi:10.3389/fpsyg.2020.00448)
Supplement: Supplementary file 1 [file Data_Sheet_1.docx]

# Supplementary material

**Supplementary Table 1.** Descriptive information for each of the images from the International Affective Picture System that we used as stimuli in the experiment. Because of spatial constraints, the stimulus descriptions were omitted from some figures (e.g., next to the dissimilarity matrices where they would otherwise be illegible), but the order of images presented in the table is the same as the order of images represented by each row of the 54 × 54 dissimilarity matrices depicted in Fig. 1A and B.

| Stimulus ID # | Description | Valence rating | Arousal rating |
| --- | --- | --- | --- |
| 1030 | Snake | 4.3 | 5.46 |
| 1202 | Spider | 3.35 | 5.94 |
| 1333 | Parrot | 6.11 | 3.17 |
| 1640 | Coyote | 6.27 | 5.13 |
| 1670 | Cow | 6.81 | 3.05 |
| 1903 | Shrimp | 5.5 | 4.25 |
| 2102 | Man Sitting | 5.16 | 3.03 |
| 2260 | Baby | 8.06 | 4.26 |
| 2300 | Lady in a Dress | 7.04 | 5.55 |
| 2441 | Staring Girl | 4.64 | 3.62 |
| 2593 | Cafe | 5.8 | 3.42 |
| 2900.2 | Happy Girl | 6.62 | 4.52 |
| 3051 | Beaten Woman | 2.3 | 5.62 |
| 3210 | Surgery | 4.49 | 5.39 |
| 3310 | Preemie | 4.37 | 5.43 |
| 3360 | Fetus | 3.78 | 5.39 |
| 3400 | Severed Hand | 2.35 | 6.91 |
| 3550 | Beaten Man | 2.54 | 5.92 |
| 4085 | Erotic Female | 5.71 | 5.77 |
| 4250 | Attractive Female | 6.79 | 5.16 |
| 4525 | Attractive Male | 6.51 | 5.17 |
| 4660 | Erotic Couple 1 | 7.4 | 6.58 |
| 4698 | Erotic Couple 2 | 6.5 | 6.72 |
| 4700 | Happy Couple | 6.91 | 4.05 |
| 5030 | Flower 1 | 6.51 | 2.74 |
| 5202 | Flower 2 | 7.25 | 3.73 |
| 5622 | Shark | 6.33 | 5.34 |
| 5780 | Lake | 7.52 | 3.75 |
| 5831 | Seagulls at the Beach | 7.63 | 4.43 |
| 5982 | Clouds | 7.61 | 4.51 |
| 6020 | Execution Chair | 3.41 | 5.58 |
| 6200 | Aimed Gun 1 | 2.71 | 6.21 |
| 6241 | Gun | 3.42 | 4.54 |
| 6250 | Aimed Gun 2 | 2.83 | 6.54 |
| 6832 | Aimed Gun 3 | 4.02 | 5.51 |
| 6940 | Tank | 3.53 | 5.35 |
| 7002 | Towel | 4.97 | 3.16 |
| 7039 | Train | 5.93 | 3.29 |
| 7042 | Hand Weights | 5.55 | 4.02 |
| 7405 | Cupcakes | 7.38 | 6.28 |
| 7512 | Chess | 5.38 | 3.72 |
| 7521 | Hospital Bed | 3.92 | 4.38 |
| 8120 | Sporty Girl | 7.09 | 4.85 |
| 8163 | Parachutist | 7.14 | 6.53 |
| 8200 | Water Skier | 7.54 | 6.35 |
| 8211 | Boat | 5.76 | 5.36 |
| 8370 | Rafting | 7.77 | 6.73 |
| 8380 | Medalists | 7.56 | 5.74 |
| 9185 | Dead Dog | 1.97 | 5.65 |
| 9420 | Dead Man | 2.31 | 5.69 |
| 9422 | Battle Ship | 4.95 | 5.09 |
| 9495 | Revolutionists | 3.34 | 5.57 |
| 9622 | Exploding Jet in Sky | 3.1 | 6.26 |
| 9908 | Car Accident | 2.34 | 6.63 |

**Supplementary Table 2.** Descriptive statistics (sum score, percentile, and T-score) of the five personality dimensions across the sample of 58 participants.

|  | Neuroticism | | | Extraversion | | | Openness | | | Agreeableness | | | Conscientiousness | | |
| --- | --- | --- | --- | --- | --- | --- | --- | --- | --- | --- | --- | --- | --- | --- | --- |
|  | Σ | %ile | T | Σ | %ile | T | Σ | %ile | T | Σ | %ile | T | Σ | %ile | T |
| μ | 22.53 | 51.77 | 49.98 | 28.67 | 51.06 | 50.09 | 33.81 | 55.19 | 51.52 | 35.48 | 72.20 | 59.67 | 33.28 | 60.82 | 53.53 |
| σ | 9.54 | 32.08 | 11.83 | 7.95 | 30.47 | 11.39 | 6.48 | 30.34 | 10.23 | 6.26 | 29.34 | 12.01 | 5.63 | 25.28 | 8.23 |

**Supplementary Table 3.** The seven models that predicted the negative cluster dispersion (in the 4-cluster solution) better than chance, as assessed by a Monte Carlo procedure. The optimal model presented in the main text is denoted here in bold.

| Predictors | β | SE | T (df) | p | p_FWER_ |
| --- | --- | --- | --- | --- | --- |
| Neuroticism | 0.381 | 0.124 | 3.085 (56) | 0.0032 | 0.019 |
| Conscientiousness | -0.388 | 0.123 | -3.146 (56) | 0.0027 | 0.016 |
| **Neuroticism**  **Conscientiousness** | **0.280**  **-0.290** | **0.127**  **0.127** | **2.202**  **2.280** | **0.032**  **0.027** | **0.014** |
| Extraversion  Conscientiousness | 0.238  -0.327 | 0.124  0.124 | -1.913 (55)  2.630 | 0.061  0.011 | 0.019 |
| Agreeableness  Conscientiousness | 0.219  -0.408 | 0.121  0.121 | -1.809 (55)  -3.363 | 0.076  0.001 | 0.021 |
| Neuroticism  Openness  Conscientiousness | 0.306  0.120  -0.290 | 0.130  0.122  0.127 | 2.354 (54)  0.981  -2.278 | 0.022  0.331  0.027 | 0.045 |
| Neuroticism  Agreeableness  Conscientiousness | 0.242  -0.172  -0.319 | 0.129  0.121  0.128 | 1.880 (54)  -1.421  -2.498 | 0.065  0.161  0.016 | 0.027 |

**Supplementary Table 4.** The optimal model for each of the clusters following from the 7-cluster solution. Note that the main findings from the 4-cluster solution (Fear/Violence and Erotic) are qualitatively unchanged, and the remaining clusters showed no association with the personality scores contained in the optimal model.

| Cluster | Predictors | β | SE | T (df) | p |
| --- | --- | --- | --- | --- | --- |
| Fear/Phobia | Extraversion | -0.212 | 0.131 | -1.620 (56) | 0.111 |
| Negative | Neuroticism  Conscientiousness | 0.278  -0.294 | 0.127  0.127 | 2.191 (55)  -2.314 | 0.033  0.024 |
| Nature | Conscientiousness | 0.123 | 0.133 | 0.931 (56) | 0.356 |
| Sports | Agreeableness | -0.113 | 0.133 | -0.850 (56) | 0.400 |
| People | Agreeableness | 0.170 | 0.132 | -1.293 (56) | 0.201 |
| Erotic | Openness  Agreeableness  Conscientiousness | -0.280  -0.291  -0.314 | 0.120  0.120  0.120 | -2.34 (54)  -2.34  -2.61 | 0.023  0.019  0.012 |
| Medical | Neuroticism | 0.207 | 0.131 | 1.58 (56) | 0.120 |

**Supplementary Table 5.** Follow-up analysis using individuals (N=41) whose affective spaces contained a 2-cluster solution (i.e., generally, a positive and a negative cluster), in which the dispersion of their individualized positive and negative clusters (i.e., *not* mapped back from the group-level median affective space) were used in the regression analysis. Here we corroborate the main findings by demonstrating that the Neuroticism + Consciensciousness model yields qualitatively similar results to those obtained from the main analysis. Despite fewer participants entering into this follow-up analysis, the directionality of the effects is maintained.

| Predictors | β | SE | T (df) | p | F (df),  p (of model) |
| --- | --- | --- | --- | --- | --- |
| Neuroticism  Conscientiousness | 0.2883  -0.31433 | 0.152  0.152 | 1.90 (56)  -2.073 | 0.065  0.045 | 6.34 (1,38)  0.0042 |

**Supplementary Figure 1.** **(Left)** Distribution of standard deviations (across participants) of the 1431 pairwise distances. On average participants’ judgments across all stimulus pairs differed by approximately 0.17 standard deviations. **(Right)** Null distribution (from 1000 iterations) of randomized average standard deviations (across participants) from the 1431 pairwise comparisons. Compared to the null distribution, the observed standard deviations of the sample tend to be far lower than chance would predicted, suggesting a degree of commonality across individual affective spaces.

**Supplementary Figure 2.** **(Left)** Distribution of Spearman correlations of individuals’ affective spaces with the group-median affective space (generated from all 101 participants). The median correlation is 0.65, and ~80% of the correlations are above 0.50. **(Right)** Distribution of Spearman correlations of the group-median affective space with the affective spaces from the 58 individuals whose personality data entered into the multiple regression analysis. The median correlation is 0.67, and ~80% of the correlations are above 0.55. These results indicate that the individuals’ affective spaces correspond relatively well to the group-median space.
